# The plastic ear and perceptual relearning in auditory spatial perception

**DOI:** 10.3389/fnins.2014.00237

**Published:** 2014-08-06

**Authors:** Simon Carlile

**Affiliations:** School of Medical Sciences and Bosch Institute, University of SydneySydney, NSW, Australia

**Keywords:** auditory spatial perception, spectral cues, auditory accommodation, auditory-motor integration, adult functional plasticity

## Abstract

The auditory system of adult listeners has been shown to accommodate to altered spectral cues to sound location which presumably provides the basis for recalibration to changes in the shape of the ear over a life time. Here we review the role of auditory and non-auditory inputs to the perception of sound location and consider a range of recent experiments looking at the role of non-auditory inputs in the process of accommodation to these altered spectral cues. A number of studies have used small ear molds to modify the spectral cues that result in significant degradation in localization performance. Following chronic exposure (10–60 days) performance recovers to some extent and recent work has demonstrated that this occurs for both audio-visual and audio-only regions of space. This begs the questions as to the teacher signal for this remarkable functional plasticity in the adult nervous system. Following a brief review of influence of the motor state in auditory localization, we consider the potential role of auditory-motor learning in the perceptual recalibration of the spectral cues. Several recent studies have considered how multi-modal and sensory-motor feedback might influence accommodation to altered spectral cues produced by ear molds or through virtual auditory space stimulation using non-individualized spectral cues. The work with ear molds demonstrates that a relatively short period of training involving audio-motor feedback (5–10 days) significantly improved both the rate and extent of accommodation to altered spectral cues. This has significant implications not only for the mechanisms by which this complex sensory information is encoded to provide spatial cues but also for adaptive training to altered auditory inputs. The review concludes by considering the implications for rehabilitative training with hearing aids and cochlear prosthesis.

## Introduction

The developing central nervous system, at first exuberant in its connectivity, is tamed and shaped by the experiences of youth to produce the fully formed and functional mature brain. This functionally plastic period of development allows the incredibly detailed connectivity of the brain to respond to the environment in which it finds itself rather than be bound and restricted by the limits of a single genetic program.

There was a time when it was believed that once organized, this developmental fluidity in the central nervous system, or “critical period,” was shut down and the mature brain was to some extent fixed in form and function. The textbook studies included those looking at the development of the visual system and the impact of optical anomalies on the subsequent development of visual cortex. To avoid the negative impact of astigmatism on subsequent visual acuity, major visual screening programs in early school age children were instituted across the Western World resulting in many small children in the school playgrounds sporting thick framed glasses.

Over the last few decades much evidence has accumulated that demonstrates that the central nervous system is far more plastic in the mature state than previously believed. Of course this makes a lot of sense when considering the environments in which mature animals live. While the body never has to again go through the explosive changes associated with its initial development, there are many changes associated with maturity and aging that still need to be accounted for to maintain a veridical perception of the environment. Moreover, some activities can have a significant impact on the structure and function of the nervous system—for instance, there is a growing body of evidence on the effects of a lifelong practice of music on some pretty basic auditory perceptual processes (for review see Strait and Kraus, 2014). Rehabilitative medicine is, to a great extent, also predicated on the functional plasticity of the mature brain.

In the context of this short review we will look at a much smaller question: how the auditory system adapts to the changes in the shape of the outer ear that occurs over a lifetime. While a small example of plasticity in the mature auditory system, one hope in pursuing this line of research is that a deeper understanding of these model systems can uncover principles that can be applied more generally. This review will conclude with some discussion of the implications of this process for training and rehabilitation, particularly in the context of hearing impairment.

## Spectral cues of the outer ear

**Figure 1:**
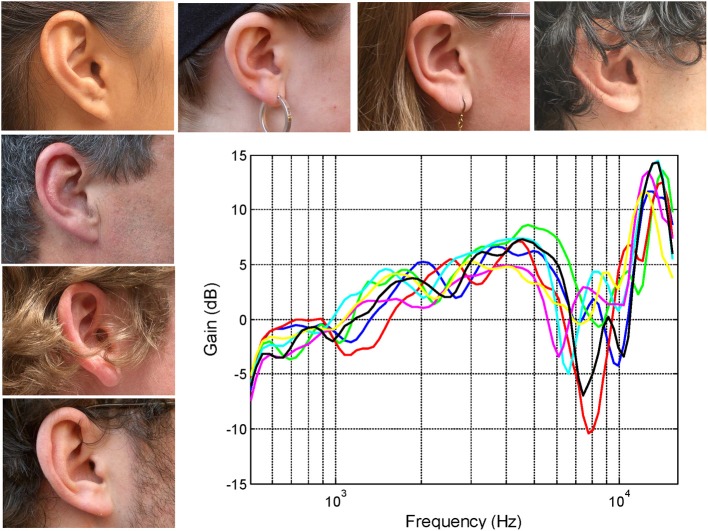
**The right ears of seven subjects together with their associated head-related transfer functions (HRTFs) recorded using a small microphone placed at the opening of the auditory canal (see Pralong and Carlile, 1994; Hammershoi and Moller, 2002)**. Note that the variations between the transfer functions remain small (<2 dB) up to around 5 kHz however, at higher frequencies, the frequencies of the prominent spectral notches and peaks results in a substantial inter-individual differences.

The shapes of the outer ear vary from person to person and it has long been argued that the precise morphology is sufficiently individualized to provide a strong form of biometric identification (see Mamta and Hanmandlu, 2013). The complexly convoluted shape of the outer ear results in a complex pattern of sound resonances and diffractions that filter the sound. Relatively small variations in the morphological characteristics of the outer ear can lead to perceptually significant differences in the spectrum of the pressure entering the ear canal (see Figure 1). So it's not just the shape of the ears that are individualized but also the spectral filtering of the sound provided to the brain. Another important acoustical property of the outer ear is that coupling of the various acoustic mechanisms with the sound field is dependent on the angle of incidence of the wave front (review Shaw, 1974). Of course this also means that the spectral filtering not only changes as a function of the relative location of the sound source but also in a manner that uniquely reflects the individual geometry of the ear.

The head-related transfer functions (HRTFs) shown in Figure 1 have been band passed from 500 Hz to 16 kHz and represent the output of the microphones placed at the opening of the ear canal for sound sources located directly in front of the listener (mid sagittal plane or midline). The precise frequencies of the sharp dips or notches reflects the complex interactions of different acoustic modes at wavelengths that are of similar size or smaller than the different morphological features of the outer ear itself. It is the differences in the distribution and interaction of these modes produced by subtle differences in the dimensions of the cavities and folds that results in the inter-individual differences of the transfer functions (see for instance Shaw and Teranishi, 1968). These subtleties are encoded in the auditory nerve despite the filtering by the cochlea (Carlile and Pralong, 1994) and are perceptually significant: For instance, it has been known for some time that listening through other peoples ears (i.e., using non-individualized spectral cues) often results in a significant degradation in sound localization performance (Wenzel et al., 1993).

In addition to the spectral cues to sound location, the auditory system utilizes the information from both ears—the binaural cues to location (see Carlile, 1996 for a review). The separation of the ears by the head means that, for sound locations off the midline, there is a difference in the time of arrival of the sound to each ear—the interaural time difference (ITD) cue to azimuth or horizontal location. Likewise, the reflection and refraction of the sound by the head gives rise to an interaural level difference (ILD), also dependent on the horizontal location of the source. The head acts as a particularly effective obstacle for sound waves when the wavelengths are smaller than the head, so ILD cues are generally thought to operate at the middle to high frequencies of human hearing. Conversely, the auditory system is most sensitive to the phase of low frequency sounds and ITD cues are particularly important for low frequencies. This observation was first made by Rayleigh (1907) and has come to be known as the duplex theory (see also Mills, 1958, 1972). These binaural cues to location, however, are ambiguous because of the symmetry of the placement of the ears on the head and can only be used to specify the sagittal plane containing the source. It is the location-dependent changes in the monaural filter functions that provide the cue to the location of the source on this so-called “cone of confusion” (Carlile et al., 2005; but see also Shinn-Cunningham et al., 2000).

**Figure 2:**
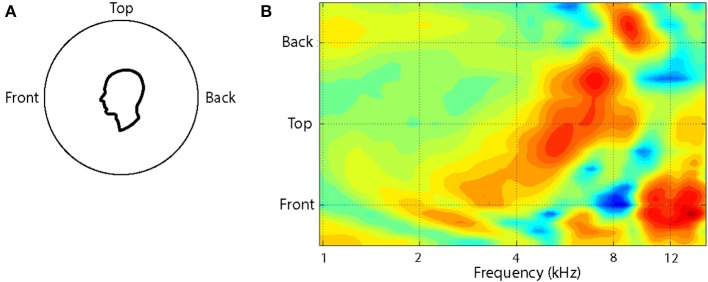
**The variation in the HRTF is shown as a function of location along a cone of confusion on the midline for the left ear of one subject (A)**. The HRTFs have been recorded at roughly 10° intervals and interpolated to provide the surface plot **(B)**. Recordings were not done for locations greater than 45° below the audio-visual horizon. The colors of the contours indicate the amplitude of the function at each frequency, location conjunction and extend from 12 dB (dark red) to −15 dB (dark blue).

The pattern of changes in the spectral cues around a single cone of confusion is illustrated in Figure 2. Plotted as a contour plot, several salient features (peaks and notches) can be seen in the HRTF for any one location but, more importantly, as the location of the source is varied from the front to the back of the listener, the frequency of these features change systematically over a range of an octave or more. For instance, when a sound source is located at the front there is a broad peak at around 4 kHz followed by a sharp notch at 8 kHz and a sharp peak at 12 kHz. When the source is located on the audio-visual (A-V) horizon but in the back, the peak is around 8 kHz and flanked by notches at 6 and 12 kHz.

While there is plenty of anecdotal evidence that the shape of the ears generally changes with age (just look at the collection of ears next time you are on public transport), the differences between ages have recently been quantified (Otte et al., 2013). Two morphological measures (ear size and conchal height) were found to be significantly different across three age cohorts: 6–11, 20–35, and >63 years. Importantly, this study also recorded the HRTFs from ears in each of the age groups. These HRTFs had substantial differences which were far larger than those seen in an age matched cohort such as those shown in Figure 1.

Some studies have look directly at the consequences of aging on sound localization performance. Reduced audibility resulting from age-related hearing loss can clearly produce a significant deterioration in performance (e.g., Noble et al., 1997). When audibility is controlled for, modest declines in performance for horizontal plane localization have been reported (e.g., Abel et al., 2000; Babkoff et al., 2002; Savel, 2009) evident principally in the front-back confusion rates (10–15%; Abel et al., 2000). In two recent studies (Dobreva et al., 2011, 2012), age-related decreases in precision (increased variance of the responses) are reported for both horizontal and vertical dimensions in the frontal hemisphere. Accounting for potential hearing loss and using different band-pass stimuli, the general consensus is that these declines represented changes in central processing of ITD and spectral cues. This is consistent with an age-related decrease in ITD sensitivity using click trains presented over headphones (Babkoff et al., 2002). Not all studies, however, have found age-related effects for horizontal localization in the frontal hemisphere (Savel, 2009; Otte et al., 2013).

In the context of the present review, while these studies generally suggest modest changes to localization performance with age, these are much less than might be expected based on the extent of the age-related change in the spectral cues produced by the changing shape of the ears (Otte et al., 2013). This suggests that the auditory system is capable of recalibrating to the progressive changes in spectral cues that occur over one's lifetime that would otherwise degrade localization performance.

## Adaptive change in the adult auditory system

Developmental plasticity is a fundamental feature of the brain. Precise neuronal interconnections and patterns of activity are sculpted by early experience to produce an incredibly complex computational system, which is tuned to its specific environment. Of interest here, though, is the level and range of plasticity in the adult auditory system.

There has been a significant amount of work looking at the plasticity of frequency tuning in the adult. Here, we are more focussed on adaptation to changing spatial cues but several general and very useful observations should be made (for an excellent and detailed review of overall auditory plasticity see Keuroghlian and Knudsen, 2007). First, the extent of plasticity seen in the adult state is not as large as that seen in the developing animal during the so-called “critical period” of development. Second, to effectively drive long-term plastic change, the stimulus generally has to have behavioral relevance such as being paired with positive or negative reinforcement or with some form of deep brain micro-stimulation (presumably triggering such reinforcement mechanisms). Third, most of these studies have focussed on auditory cortex and generally found that cortical tuning can be adjusted independently for a range of parameters including frequency, level, and temporal selectivity. Fourth, previous training induced changes can be preferentially selected depending on the behavioral context of the task at hand (see also in particular Fritz et al., 2003, 2005; Keating et al., 2013).

Relatively fewer, but no less important studies, have examined the plasticity induced by changes to the auditory spatial localization cues (review Wright and Zhang, 2006). The simplest method of varying the binaural cues has been to insert an ear plug in one ear (Bauer et al., 1966; Florentine, 1976; Musicant and Butler, 1980; Butler, 1987; Slattery and Middlebrooks, 1994; McPartland et al., 1997; Kumpik et al., 2010). This approach produces relatively straight-forward changes in the sound level in the plugged ear although the effects on ITD are more complex and dependent on the conditions of the plugging (e.g., Hartley and Moore, 2003; Lupo et al., 2011).

Before proceeding with a more detailed discussion of these results, an important methodological issue needs to be considered. When studying the binaural cues to sound location, the stimulus of choice is often restricted in frequency range—low frequencies for ITD studies and middle to high frequencies for ILD studies. This reflects the different frequency ranges that these cues are thought to operate over (the so called duplex theory of localization processing discussed above). On the other hand, the greater bulk of the research examining auditory localization has used broadband noise as the stimulus. This is motivated principally by the fact that such stimuli contain the full range of acoustic localization cues and in particular, the spectral cues are necessarily dependent on a broad frequency range. An important distinction therefore is that stimuli with a relatively restricted frequency range are designed to probe the contributions of a single cue while a broad spectrum stimulus will provide the full range of acoustic cues to a sounds location.

Returning to the ear plugging experiments, when sound localization performance was measured immediately after inserting the ear plug, performance was significantly reduced and then recovered to a certain extent over a period of days [Bauer et al., 1966 (2–3 days); Kumpik et al., 2010 (~7 days)]. No recovery was found for shorter 24-h periods of plugging (Slattery and Middlebrooks, 1994). Studies examining ILD sensitivity with one ear plug are more mixed with one demonstrating adaptive change in ILD sensitivity (Florentine, 1976) and another finding only modest changes in a subset of listeners (McPartland et al., 1997) and another reporting no evidence of binaural adaptation (Kumpik et al., 2010).

Other studies have modified the binaural ITD cue using a hearing aid in one ear (Javer and Schwarz, 1995), a “pseudophone” (an arrangement of 2 microphones feeding into two ear pieces that could be manipulated independently of the head orientation: Held, 1955) or headphones presenting stimuli in virtual auditory space (sounds filtered with HRTFs but with changes in the normal ITDs: Shinn-Cunningham et al., 1998). Using localization performance as the metric these studies all report initial biases in localization consistent with the binaural change and subsequent reduction in bias following several (3–5) days (Javer and Schwarz, 1995), several (~7) hours of exposure (Held, 1955) or even repeated, relatively short (2 h) training sessions repeated over 2–6 weeks (Shinn-Cunningham et al., 1998), although adaptation was never complete.

Importantly, the work of Kumpik et al. (2010) mentioned above was one of the few studies that demonstrated adaptive change in auditory localization following ear plugging but intriguingly, found no changes in binaural sensitivity in parallel with those changes. Rather, these authors attribute the adaptive change to a relative reweighting of the binaural and monaural spectral cues to location (see also Kacelnik et al., 2006; Van Wanrooij and Van Opstal, 2007). The range of difference in the results of the previous studies could then be explained by reweighting of the different cues available in each study or other practice effects (Musicant and Butler, 1980; Butler, 1987).

**Figure 3:**
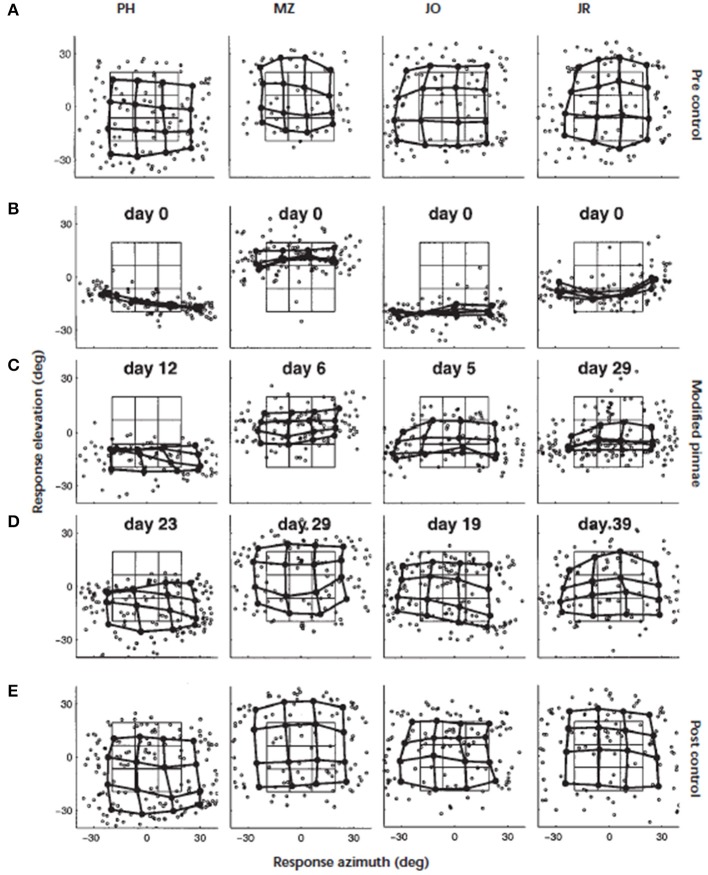
**Adaptation to altered spectral cues**. Localization behavior of four subjects (from left to right) before, during, and immediately after the adaptation period. Day 0 marks the start of the adaptation experiment. The panels show, for each subject, the individual saccade vector endpoints in the azimuth–elevation plane (symbol °). In addition, the saccade vectors were also averaged for targets belonging to similar directions by dividing the target space into 16 half-overlapping sectors. Averaged data points (solid circle) from neighboring stimulus sectors are connected by thick lines. In this way, a regular response matrix indicates that the subject's saccade endpoints capture the actual spatial distribution of the applied target positions. The target matrix, computed in the same way as the saccade matrix, has been included for comparison (thin lines). **(A)** Results of the preadaptation control experiment on day 0, immediately preceding the application of the molds. **(B)** Localization responses immediately after inserting the molds (day 0). Note the dramatic deficit in elevation responses for all subjects. **(C)** Results during the adaptation period after 12 (PH), six (MZ), five (JO), and 29 (JR) days of continuously wearing the ear molds. **(D)** Results near the end of the adaptation period. Stable and reasonably accurate localization behavior has been established in all subjects. **(E)** Results of the control condition, immediately after removal of the molds. All subjects localized sounds with their original ears equally well as before the start of the experiment several weeks earlier. Figure 2 from Hofman et al. (1998).

This turns our focus to the monaural cues, which in ecological terms, are the more likely cues to be modified by the progressive changes in pinna shape over a lifetime. Around the turn of the twentieth century, Hofman et al. (1998) demonstrated that the adult auditory system was able to accommodate to substantial changes in the filter functions of the outer ears. Elevation localization was significantly disrupted when the HRTFs of human listeners were modified by inserting small molds in the concha (Figure 3). For the four listeners who wore the molds continuously, elevation localization improved significantly over periods ranging from 19 to 39 days. Furthermore, once the molds were removed, localization performance was the same as their performance before wearing the molds. This indicated that accommodation to the “new” cues did not interfere with representation of the “old” cues. The changes in spectral cues induced by the molds were both substantial and abrupt and unlike the slow, progressive changes that would occur through life. Nonetheless, this was a critical study that demonstrated the adaptive capability of the adult auditory system to changes in the shape of the outer ear.

Although there were only four subjects in that study, two other interesting observations can be made. First, there were significant individual differences in the rate of accommodation—the shortest being 19 days and the longest twice as long at 39 days. Second, the localization performance of three subjects approached that of pre-mold baseline, while the fourth subject fell somewhat short. One inter-subject variable may have been different environmental opportunities to relearn their new filter functions over the accommodation period. In ferrets (Kacelnik et al., 2006) and humans (Kumpik et al., 2010), King and colleagues demonstrated that unilateral ear plugs disrupted the azimuthal sound localization as discussed above but that, over a period of seven or more days, performance improved with training. Although an ear plug principally disrupts the binaural cues it will also produce distortions to the spectral cues in one ear, however, the principal point of interest here is the effect of experience on the accommodation. The amount of training *per se* did not appear to be a principal driver as performance improvements were only evident when the training was spread over the 7 days rather than simply delivered as a single large block of training.

A second inter-subject variable in the Hofman et al., study could have been the magnitude of the changes to HRTFs produced by the molds. Consistent with this was the later finding that accommodation to monaural ear molds was dependent on the magnitude of the difference in the spectral cues between the bare ear and the mold ear (Van Wanrooij and Van Opstal, 2005). An overall similarity index (SI) was calculated from the standard deviations of the correlations between the HRTFs recorded from the anterior midline, with and without the molds. For 8 of 13 subjects, low similarity appeared to induce accommodation whereas the remaining five subjects, with only moderate differences between the mold and bare ear HRTFs, demonstrated oscillatory patterns in performance over the accommodation period rather than any progressive improvement.

In summary, modifying the binaural inputs by plugging one ear produces an acute decrease in auditory localization performance that recovers to some extent over a small number of days. This recovery does not seem to be accompanied by an adaptive variation in sensitivity to the binaural cues to location. Relatively subtle modifications to the monaural spectral cues also produce an initial reduction in localization performance in the elevation domain (on the cone of confusion) that also generally recovers to some extent over a period of 2–4 weeks. In the case of the ear plug, it is likely that the monaural cues provided by the plugged ear are also disrupted and the relatively rapid performance recovery has been attributed to a reweighting of the location cues to initially prioritize the veridical monaural cue provided by the unplugged ear. The differences in the accommodation times for the unilateral plugging compared to the bilateral molds is consistent with the idea that different processes might underlie the localization performance improvements in each case.

## Effects of vision on auditory spatial tuning

The role of visual input in guiding the development of the auditory spatial representation in the mammalian midbrain nucleus, the superior colliculus (SC) and its homolog the optic tectum of the barn owl, is well-documented. This is a particularly convenient nucleus to examine these interactions because of the topographic representation of auditory space and its spatial correspondence with the retinotopic visual representation. In an early developmental study using neonatal ferrets, a strabismus was induced in the one eye by cutting an extra-ocular muscle. The resultant shift in the visual representation in the SC induced a compensatory shift in the developing auditory representation, which maintained alignment of the two modalities (King et al., 1988). Similarly, shifting the visual field of the developing barn owl using optical prisms fixed in front of the eyes produced a similar shift in the auditory map in the optic tectum (Knudsen and Brainard, 1991). A range of other experimental manipulations have further underscored this developmental interaction (recent review: King, 2009).

However, vision is not necessary for the development of auditory spatial perception. Congenitally blind individuals are able to localize the source of a single sound with equal or even superior levels of performance compared to sighted individuals (e.g., Roder et al., 1999). There is, however, some evidence that congenitally blind localizers may be impaired perceiving more complex spatial relations between multiple sound sources (Gori et al., 2014).

There are also many examples of real-time audio-visual interaction in sound localization: Accuracy can be improved if the target is also visible (Shelton and Searle, 1980); Spatial disparities in synchronous audio-visual stimuli can result in the auditory location perceived as closer to the visual location (visual capture or the ventriloquist effect: e.g., Bertelson and Radeau, 1981); The ventriloquist after-effect can persist for minutes (e.g., Radeau and Bertelson, 1974; Woods and Recanzone, 2004).

Over a slightly longer time frame, conditioning the adult visual systems using distorting lenses for 3 days can lead to some compensatory distortion of auditory space (Zwiers et al., 2003). In a series of experiments using adult barn owls, Knudsen and colleagues examined the impact of shifting the visual field on the ITD tuning of neurons in the optic tectum. Prism lenses of increasing strength were used to incrementally shift the visual field. A progressive and corresponding shift in ITD tuning maintained the audio-visual coincidence in the neural representation (Linkenhoker and Knudsen, 2002). This incremental approach to retuning produced a five-fold greater change in neural tuning compared to a single large displacement of the visual field. Interestingly, owls that had accommodated progressively were able to later rapidly accommodate to a single large shift. In another experiment where owls were fitted with displacing prisms, hunting for live prey produced five-fold greater adaptive shift in ITD tuning in the optic tectum compared to owls that, under the same conditions, were fed dead mice. On the one hand this highlights the importance of bimodal stimulation in this accommodation (live mice are coincident auditory and visual targets) and a role for attention, arousal and behavioral relevance (reward). On the other hand, the audio-motor interactions involved in capturing live prey are far more complex than that for dead prey—this is a theme to which we will return in more detail.

## Visual input and accommodation to perturbed spectral cues

The first demonstration of adult auditory plasticity to perturbations in the spectral localization cues, discussed above (Hofman et al., 1998), used eye pointing to indicate the perceived location of a sound source. As a consequence, the possible range of locations was limited to ±30° from directly ahead. In a later study, the same group looked at the effects of monaural molds using eye pointing and this time the range of possible locations was ±70° (Van Wanrooij and Van Opstal, 2005). For locations within the visual field, any mismatch between the perceived auditory and visual locations of a sounding object could be used as a teacher signal as the auditory system recalibrates to the new spectral cues. This poses the interesting question as to whether the auditory system is even capable of retuning the spectral cues to locations outside the visual field in the absence of simultaneous visual input. Concurrent audio-visual inputs are not available for locations outside the visual field so, if the auditory system is able to accommodate to cues pointing to these locations, we might expect a different mechanism to be operating.

**Figure 4:**
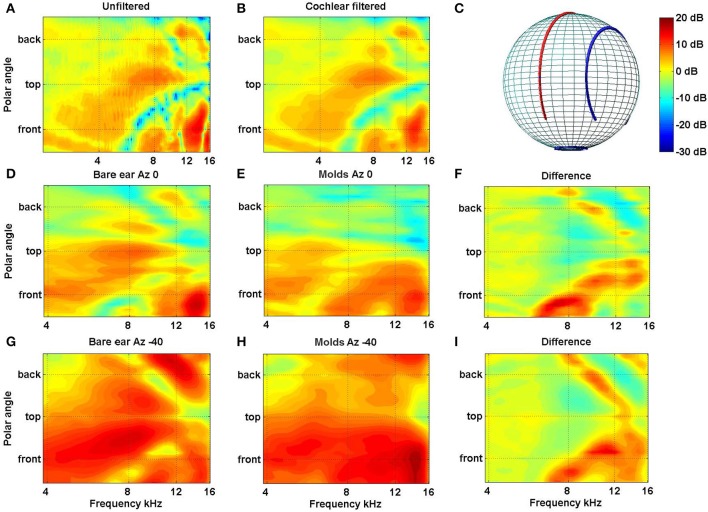
**(A)** Filter functions of the left ear of one subject are plotted for the midline cone of confusion before and **(B)** after passing through a cochlear filter model. The features in **(B)** indicate that, despite the frequency filtering and spectral smoothing produced by the cochlear, substantial spectral features are preserved within the auditory nervous system. Filter functions for the left ear of a different subject are plotted for the midline [**D–F**: Azimuth 0°, cf. red line in **(C)**] and 40° off the midline [**G–I**: Azimuth −40°; cf. blue line in **(C)**] are plotted without molds **(D,G)** and with molds **(E,H)**. The data have been smoothed, as above, using the cochlear filter model. The differences between the bare ear and mold conditions for both lateral angles are plotted in **(F,I)** (Data from Carlile and Blackman, 2013).

In a recent study in our laboratory we looked at the extent and rate of accommodation to new spectral cues for locations inside and outside the visual field (Carlile and Blackman, 2013). As in previous studies we used small bilateral ear molds to distort the spectral cues provided by the outer ear. The acoustic impact of the molds are shown for the left ear of one subject (Figure 4) and crucially, the molds can be seen to have modified the spectral cues for the posterior as well as the anterior hemispheres [see in particular panels **(F,I)**].

**Figure 5:**
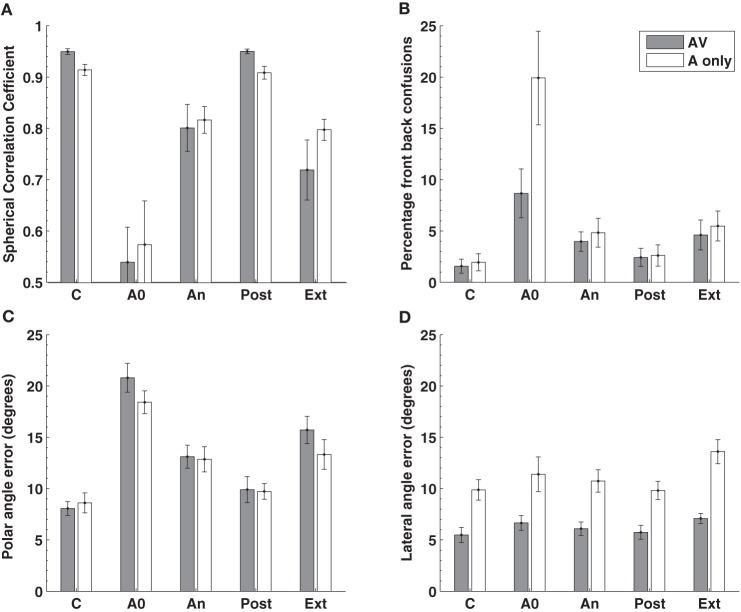
**Localization performance before, during, and after an accommodation period where spectrally distorting pinna molds were worn**. Localization performance was measured using the **(A)** spherical correlation coefficient, **(B)** the percentage of front-back confusions, **(C)** the polar angle error (elevation error on the cone of confusion) and the **(D)** lateral angle (azimuth) error. The experimental manipulation is shown on the X-axis: C, control or baseline performance without the mold; A0 effect of acute placement of the mold; An, performance at the end of the accommodation period (mean 40.5 days); Post, performance immediately after removing the molds at the end of accommodation; Ext, performance on reinsertion of the molds more than a week after the end of accommodation. The gray bars represent data obtained from the audio-visual region of space (±70° from the midline) while the open bars represent data from the audio-only region outside these limits. Figure 2 from Carlile and Blackman (2013).

In contrast to previous studies we examined localization performance for 76 sound locations equally spaced around the listener. Insertion of the molds produced, on average, a seven fold increase in the number of front-back hemispheric confusions and a doubling of the polar angle (elevation) error (Figures 5B,C, 1st cf. 2nd columns). Subjects wore the molds continuously for 32 or more days (average 40.5 days) and showed an improvement in performance toward pre-mold (control) values (Figures 5B,C, An cf. C). Critically, post accommodation (An) none of the performance parameters demonstrated a difference between locations within the audio-visual field [defined in this study as ±70° about the point directly ahead (gray bars)] and the audio only region [the rest of the sphere surrounding the listener (open bars)].

This indicates that (i) the system was able to accommodate to, or remap, new spectral cues in the absence of concurrent visual information and (ii) that the extent of accommodation was identical for both regions of space. That study also went on to examine the time course of accommodation and also found no differences in the rate of accommodation for the audio-visual compared to the audio-only regions of space. These latter findings are consistent with the idea of a single underlying process for both regions rather than one process that relies on vision and another that doesn't.

Removing the molds at the completion of the accommodation period resulted in an immediate return to control levels of performance (Figure 5, C cf. Post). This confirms the previous observation in a smaller group of subjects (Hofman et al., 1998) and indicates that despite more than a month of exposure and accommodation to the “new” spectral cues, the brain's representation of the “old” spectral cues was intact. Subjects also returned a week or more after the accommodation period, over which time they had not been wearing their molds. At this time, localization performance was tested with the molds reinserted and was not different from their accommodated performance (Figure 5, An cf. Ext). This suggests that following acquisition of the “new” cues, the auditory system was able to retain this mapping despite being chronically exposed once again to the “old” cues.

## Non-auditory inputs in sound localization

A primary survival advantage provided by the auditory system is the detection and accurate localization of sources outside the listener's visual field. It therefore makes sense that the auditory system is able to effectively accommodate to changes in auditory cues that point to locations both inside and outside the visual field. At a minimum, maintaining the accurate calibration of the spectral cues resolving front from back on the cone-of-confusion would be essential to manage appropriate responses for example the approach of a predator. These data, together with the fact that congenitally blind individuals can localize sounds, raise the obvious question “if not vision, then what?”

In answering that question we need to spend a little time looking at how we got here. Much of the work on auditory localization over the last century or so has followed in the excellent footsteps of Rayleigh (1907) and examined in some detail the relative contributions of the different acoustic cues to localization processing (reviews Middlebrooks and Green, 1991; Carlile, 1996; Carlile et al., 2005; Letowski and Letowski, 2012). On the one hand, these efforts have given us a good understanding of how we derive spatial information from the acoustics at each ear. On the other hand, the focus has primarily been on a single static sound source and speaks little to the manner in which this information is integrated with other non-auditory information to drive or guide action. The focus has largely been on pure tone or broadband noise stimuli presented under anechoic conditions and in silence and only recently have more real world stimuli such as speech (e.g., Best et al., 2005) been used in combination (Kopco et al., 2010) and in reverberant settings (Shinn-Cunningham et al., 2005; but see also Hartmann, 1983).

One important and related question is the spatial coordinates used in auditory localization processing. The ears of humans are relatively immobile and symmetrically placed on the head so that the coordinates of the acoustic cues to location are head-centered. In order to perceive and interact with the spatial location of sound sources, the location of the head with respect to the body needs to be taken into account. These sorts of questions have uncovered a wide range of important non-auditory influences on auditory localization performance.

In one study, using a sequence of an auditory then a visual stimulus, subjects first had to orientate to the (later) visual target and then to the (earlier) auditory stimulus. Although shifting the head to the later visual stimulus would change the head-centered coordinates of the auditory stimulus, subsequent orientation to the earlier auditory stimulus was still highly accurate (Goossens and Van Opstal, 1999). This suggests that the earlier auditory target was encoded in a body centered, rather than a head-centered, frame of reference. This study also suggested that head orientation had some influence on the localization of auditory target under static conditions. Another study using an ILD adjustment task, reported that shifts in the perceived midline of static stimuli were influenced by the right-left orientation of either the head or the eyes with the head fixed (Lewald and Ehrenstein, 1998). As the influence of both eye position and head position were about the same, they canceled out when the eyes were fixated on the auditory target, regardless of the head position. Similar results were obtained for both horizontal and vertical dimension using a laser pointing task to actual sound sources (Lewald and Getzmann, 2006). More recent detailed work has demonstrated that the spatial shift induced by eye position occurs in the absence of a visual target and also induces a shift in the perceived midline (Razavi et al., 2007; Cui et al., 2010). Vestibular stimulation has also been shown to influence the auditory spatial perception in the absence of change in the relative posture of the head (Lewald and Karnath, 2000; Dizio et al., 2001). This is far from an exhaustive review of this literature but the emerging picture suggests that a range of non-auditory inputs relating to the relative location of the head and eyes are also integrated with the acoustic cues to encode spatial location in body centered coordinates.

There are a range of sources of information about motor state including motor efference copy, proprioception and vestibular and visual information, all of which provide a dynamic, real time stream of data. If the head-eye position effects on auditory localization share the same mechanisms underlying similar effects in visual localization (see Hallet and Lightstone, 1976) then efference copy information regarding head position may be playing the driving role (see Guthrie et al., 1983). In a recent study in our laboratory, we have been looking at the ability to track a moving auditory stimulus using nose pointing (Leung et al., 2012). Listeners with schizophrenia, where motor efference copy mechanisms are thought to be severely disrupted (Ford et al., 2008), show significant deficits in this audio-motor tracking task (Burgess et al., 2014). In contrast, these patients did not show any deficits in the perception of the velocity of a moving auditory target *per se*, perceptual judgments that did not involve head movement. A role for motor efference in auditory spatial perception is also consistent with the distortions of auditory space that occur with rapid head saccades (Leung et al., 2008).

Whatever the mechanism, these experiments demonstrate that information about the motor state strongly influence the analysis of the acoustic information underlying the perception of space. From this perspective, sound localization is transformed from being a problem of the computational integration of the binaural and monaural acoustic cues to the static location of a sound source (a remarkable enough feat in itself) to a highly dynamic process involving a number of coordinate transformations and the disambiguation of source and self-motion. Consistent with this idea, it has been known for some time that, when a sound stimulus is of a duration that permits small head movements, multiple sampling of the sound field increases the localization performance, particularly in the context of resolving front-back confusions (Wightman and Kistler, 1999; see also Brimijoin and Akeroyd, 2012). More recently, the integration of self-motion information has also been shown to play an important role in the perception of an externalized sound source (Brimijoin et al., 2013).

At a theoretical level, it has recently been demonstrated that an auditory spatial representation can be established purely on the basis of audio-motor information. In a very important modeling study Aytekin et al. (2008) described a machine learning system that was able to construct a veridical representation of directional auditory space based on knowledge about (i) its own orientation movements and (ii) the auditory consequences of that movement. Put simply, their system made an “orientation movement” relative to some internal coordinate system and was then provided with two HRTFs that corresponded to that orientation. Over many pairs of movements and samples, the system built up an ordered list of the HRTF pairs that corresponded to the many different possible orientations from which the HRTFs were originally recorded. Their model was equally successful using human HRFTs taken from the CIPIC data base (Algazi et al., 2001) and on a collection of bat HRTFs. Other sensory-motor models of auditory localization have been subsequently developed (e.g., Bernard et al., 2012). Such models may provide a basis for understanding how auditory localization develops in the congenitally blind or how the mature auditory system is able to retune to spectral localization cues in the absence of visual input.

## The effects of sensory-motor feedback on auditory accommodation

In the previous work showing accommodation to ear molds, we and others have found that there is a significant range of individual differences in both the extent and range of accommodation. Some subjects appear to asymptote in performance after a couple of weeks of wearing the molds, while others continue to improve over 4 or 5 weeks. Similarly, while most subjects show performance changes that approach their pre-mold, control levels, others improve far less (Hofman et al., 1998; Van Wanrooij and Van Opstal, 2005; Carlile and Blackman, 2013). Such difference could reflect individual differences in the capacity of the auditory system to adapt, although, given the relative homogeneity of the subject pool we feel this is unlikely. It is more likely, the inter-subject variance in accommodation could be caused by (i) different experiences and learning opportunities during the accommodation period and/or (ii) by differences in the acoustic distortion provided by the subjects' molds.

Taking the latter case first, acoustically related accommodation changes could result from differences in the extent of the distortion of the spectral cues produced by each mold. While the molds all looked fairly similar in size and shape, this is consistent with the large acoustic impact of relatively small differences in the sizes and shapes of normal outer ears (Figure 1; e.g., Shaw, 1974; Carlile and Pralong, 1994; Carlile, 1996). This could influence the size of the step change from the “old” to “new” spectral cues which may play a role in triggering and/or sustaining accommodation (Van Wanrooij and Van Opstal, 2005). In addition, the extent of performance improvements due to accommodation is also likely to be dependent on the spatial quality of the residual cues. For instance, near complete abolition of directionally dependent cues will provide very little acoustic spatial information for the auditory system to accommodate to.

We have recently completed an accommodation study where we first attempted to control for variations in the extent of spectral disruption produced by the mold and second, then focussed on the accommodation effects of training using sensory-motor feedback to source location. We found that a mold that filled the ear 40% by volume produced significant changes in localization performance when first inserted but retained elevation dependent acoustical changes in the frequencies of prominent spectral peaks and notches of the order an octave. We fitted these “standardized” molds to four groups of subjects and measured localization performance in response to different training regimes (Carlile et al., 2014).

The focus of the training regimes was to provide different levels of sensory and motor feedback each day of accommodation in addition to the subject's normal daily experiences. Given the theoretical modeling of the role of audio-motor feedback discussed above (Aytekin et al., 2008), we wanted to ensure a strong audio-motor component in the training regime. As before, localization testing and training was done in a darkened anechoic chamber. The first group received no performance feedback (Control) and just did three blocks of localization testing each day of accommodation; the second group received only visual feedback using a LED illuminated on the stimulus loudspeaker following each localization trial (Visual); the third group received visual and audio feedback where following each localization trial, the target loudspeaker pulsed at a rate inversely proportional to the nose-pointing error [Audio Visual Sensory Motor group (AVSM)]. In an attempt to maximize the audio-motor feedback, subjects were encouraged to explore the space around the target by moving their heads and to minimize the pointing error using this audio feedback before registering their corrected response; the fourth group used the AVSM paradigm with the room lights turned on during training. This provided subjects with an additional allocentric frame of reference over and above the body centered frame of reference provided by the endogenous audio-motor information [AVSM-Frame of Reference (AVSM-FOR)].

**Figure 6:**
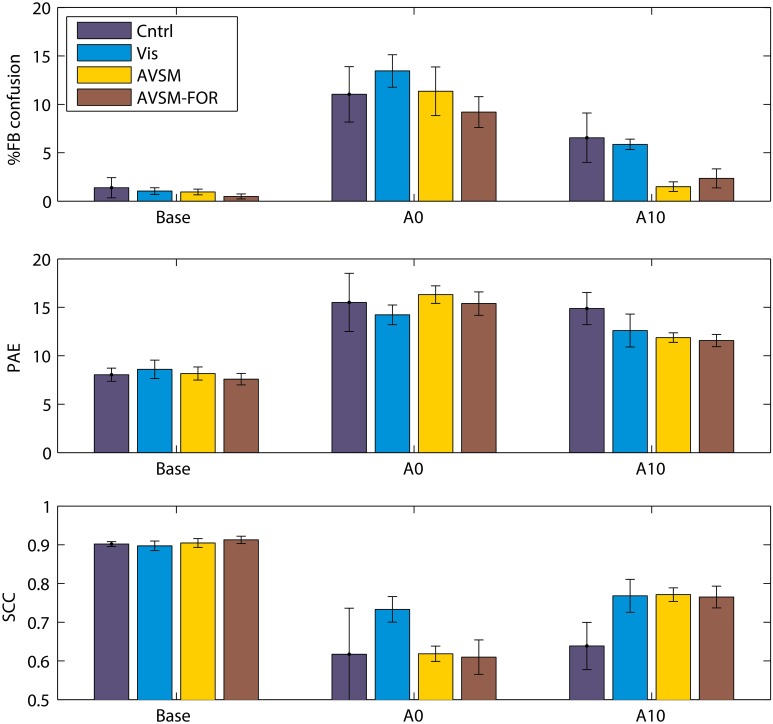
**The effects of training on accommodation to ear mold**. Base: performance before accommodation with bare ears; A0: Performance on acute exposure to the mold; Acm10: performance following 10 days testing with feedback. PAE, Polar angle error; SCC, Spherical correlation coefficient. Data from Carlile et al. (2014).

In contrast to previous studies, when compared to baseline, the acute effects of the molds were very similar for each group (Figure 6, Base cf. A0), confirming that the standardization of the spectral perturbation had to a large extent been successful. The difference in the feedback regimes can be seen most clearly in the front-back confusion rates by the tenth day of accommodation (Figure 6, top panel A10). While there was some improvement in the Control and Visual groups the most significant changes were for the groups receiving AVSM feedback. Similar improvement can also be seen with the elevation errors (PAE) although visual feedback alone was not significantly different to the AVSM feedback. The allocentric frame of reference (AVSM cf. AVSM-FOR) did not seem to confer further advantage, consistent with the idea that spatial location is coded in body-centered coordinates that does not require an external reference frame (Goossens and Van Opstal, 1999). Looking across the 10 days of accommodation it also appeared that AVSM feedback regimes produced a much quicker asymptote in performance at around 5–6 days (data not shown).

Undoubtedly accommodation was occurring in the absence of any feedback-based training regime, presumably on the basis of the daily experience of the subject outside the laboratory, just as in the previous studies using ear molds. By contrast, however, AVSM feedback, in particular, resulted in an increased rate of and greater extent of accommodation. Three other studies have employed similar forms of sensory-motor feedback in assisting listeners to accommodate to non-individualized HRTFs used in virtual auditory displays (Zahorik et al., 2006; Parseihian and Katz, 2012; Majdak et al., 2013). Interaction with the sound objects in the display was a key part of each study and some improvements in front-back confusion rates were generally found after relatively short periods of training (Zahorik et al., 2006; Parseihian and Katz, 2012) however front-back confusion rates were still significantly higher than performance seen for subjects localizing in the free field with their own ears. With a longer period of training (21 days of 2 h sessions) improvements in both front-back confusion rates and elevation errors were reported (Majdak et al., 2013). A very interesting outcome of these studies, when compared to those employing molds, is that the auditory system appears to be able to accommodate to a different set of cues even though it does not experience a consistent exposure to the new cues over the full period of accommodation. In the case of the virtual display studies, as soon as the training session is complete the listeners are then listening through their own ears. By contrast, the molds listeners are encouraged to keep them in their ears during all waking hours (except when swimming or bathing).

## Conclusions and implications

Investigations of auditory adaptation to changes in the spectral inputs have highlighted a number of interesting and important aspects of auditory localization processing. It seems likely that localizing sounds in the real world involves a range of non-auditory inputs, which may also be co-opted in the process of accommodating to changes in the auditory cues. Firstly, despite the early focus on the visual system's involvement in the development of auditory representation, it appears that visual input is not necessary for auditory accommodation to cue changes in the mature animal. There is a growing body of evidence that the motor state has an impact on the perception of auditory location. Again, the ecological problem of sound localization of even a single source is best characterized as a dynamic process involving the (i) transformation of the head-centered, acoustic cue coordinates to body-centered spatial coordinates and (ii) the disambiguation of source and self-motion. On-line information regarding motor state is critical to such processing—whether this represents motor efference copy information (as is the case for the visual system) or proprioceptive feedback or a combination of the two is very much an open question. Regardless of the mechanism, motor state information has also been shown to be, theoretically, sufficient to establish a veridical representation of auditory spatial information.

In this light, the demonstrated capacity to recalibrate to acoustic cues that point outside the immediate visual field and the impact of audio-motor training regimes on accommodation should not be that surprising. The range of individual differences seen in previous spectral accommodation studies using ear molds could also reflect the audio-motor training opportunities available to the individual. This of course raises the question of the capacity of such training regimes to promote, accelerate or complete accommodation to other forms spectral input changes including the application of hearing aids, changes to a hearing aid's processing or to the enhancement of the acoustic cues to location by the hearing aids. The role of attention and motivation in the perceptual learning of the altered spectral cues is likely to be a critical element in the success of any training regime (see Amitay et al., 2006; McGraw et al., 2009; Molloy et al., 2012). Although we have not been able to examine this literature in the course of this review there has also been much work in perceptual learning in the visual system (e.g., see Shams and Seitz, 2008; Deveau et al., 2014) that can also inform the development of effective auditory spatial training paradigms.

A recent study of the HRTFs obtained through different hearing aid styles (e.g., Completely in Canal vs. Behind the Ear) demonstrated substantial spectral cue differences associated with different form factors (Durin et al., in press). Moving from one hearing aid style to another would be expected to be the equivalent at least of fitting ear molds as described above. Real time signal processing also provides the potential for enhancement of spectral or other cues to spatial location which could aid in localization (Majdak et al., 2013) and/or the intelligibility of speech in noise (Jin et al., 2006). Clearly these kinds of enhancements would require the auditory system to accommodate to substantial changes in the localization cues and efficient means of driving such accommodation would aid substantially in their utility. Of course, the most substantial accommodation required of the auditory system follows the fitting of a cochlear prosthesis, which requires many months or years of training. The challenge here is to broaden research and discover whether the audio-motor interactions underlying the accommodation to spatial cues can also be applied more broadly to spectrally-temporally complex signals such as speech.

### Conflict of interest statement

The author declares an interest in a company, VAST Audio Pty Ltd. that is seeking ways in which auditory perceptual processes can be applied to hearing aid developments and auditory rehabilitative training to aid in solving the cocktail party problem in the hearing impaired. No third party funds, other than the competitive grants provided by the Australian Research Council have been provided to support this research.
